# Expectations about check-up examinations among Swiss residents: A nationwide population-based cross-sectional survey

**DOI:** 10.1371/journal.pone.0254700

**Published:** 2021-07-21

**Authors:** Laura Diaz Hernandez, Stéphanie Giezendanner, Roland Fischer, Andreas Zeller

**Affiliations:** Centre for Primary Health Care, University of Basel, Basel, Switzerland; University of Tripoli, LIBYA

## Abstract

**Introduction:**

General health check-up examinations in asymptomatic adults have not been shown to be beneficial. Instead, opportunistic prevention during regular primary care consultations is most cost-effective and recommended. The study aimed to elucidate the expectations about check-ups of the general Swiss population.

**Methods:**

A nationwide cross-sectional telephone survey was conducted in a representative sample of the population, stratified by sex, age, and language in November 2019.

**Results:**

Data of 1077 respondents were analysed. Mean age was 45 years (range 18 to 89), and 51% were female. Overall, 40% of respondents expected to have check-up examinations (yearly: 41.6%), and 42% expected opportunistic prevention. Most expected check-up interventions were sex-specific such as mammography (89% of women), Pap smear test (89% of women), and blood test of prostate-specific antigen (81% of men). The least favoured ones related to counselling (tobacco: 27%; alcohol abuse: 29%). Most significant predictors of positive check-up expectations were being male (OR = 1.45, CI: 1.02–2.05 *P* = 0.04)), age between 45 and 59 years old (OR = 2.03, CI: 1.27–3.23, *P* = 0.003 vs. 18 to 29 years), having a degree from professional (OR = 1.73, 95% CIs: 1.11–2.69, *P* 0.015) or, middle school (OR = 1.99, 95% CIs:1.04–3.78, *P = 0*.*037*) or university (OR = 1.66, 95% CIs: 1.06–2.61, *P< 0*.*001*, vs. secondary school) and the more importance attributed to regularly checking one’s health (OR = 2.12, 95% CIs: 1.70–2.36, *P* < 0.001)

**Conclusions:**

Almost half of the population expected to have mostly yearly check-up examinations in addition to regular care, which is in contradiction to recommendations. This behaviour impacts the rational use of health care resources and must be considered by physicians and given the active role of patients in the health care system.

## Introduction

A comprehensive annual physical examination or check-up is a medical preventive intervention performed on an asymptomatic person to assess the general health state. Under the idea that check-ups would reduce disease, they became standard practice in many countries during the last century and were strongly encouraged to the general population [1, 2]. This mindset seems to have persisted across time, even though current evidence suggests that check-ups do not reduce morbidity or subsequent medical care services use as expected [3], and thus guidelines favour abandoning this practice. Instead, *opportunistic prevention* is recommended, as it is deemed to be more beneficial [3]. Opportunistic prevention includes anticipatory care (health promotion and disease prevention), case-finding (screening tests to detect early disease or risk factors for disease), and continuity of care (healing, chronicity, or death) [4]. Opportunistic prevention takes place during a regular care visit to the physician for a chronic or an acute illness that needs to be dealt with beforehand. The consultation offers an opportunity to discuss and include preventive interventions [4, 5], which must be tailored to the patient based on sex, age, and individualized risk. There is no unique strategy that would fit everyone, but preventive interventions should include counselling, immunization, and physical examination according to the patient’s situation.

Physicians in Switzerland have several sources to access preventive evidence-based medical recommendations to which they should adhere [6–8]. These recommendations are founded on the US Preventive Services Task Force [9, 10] and are adapted to the Swiss context and supported by the Swiss Society of General Internal Medicine and the College of Family Physicians. A table summary can be found on the EviPrev website (https://eviprev.ch/downloads/). Patients, however, are most likely unaware of them and left to rely on their uninformed expectations to discuss and accommodate their physicians’ recommendations.

Patients’ expectations about medical prevention might be driven by several factors including the overestimation of the positive effects of some preventive interventions [11], the illusion of reassurance that prevention gives [12], the lack of individual assessment of potential benefits and risks, the advances in technology, the abundance of medical tests available, as well as the cultural belief that more is always better. A study in the US concluded that the population had a high desire for comprehensive annual check-ups and that this desire was sensitive to charges [13]. More recently, studies in Portugal showed that patients overestimated the benefits of preventive interventions and had a tendency to overuse resources as they believed they should use a great number of services annually [14, 15].

Those studies let us conclude that public expectations do not seem to be aligned with current medical recommendations. This fact bears utmost importance in a health system with a “shared medical decision-making model” where decisions are discussed and shared between physicians and patients [16]. Patients can express their opinions and preferences and have the option to ask questions about medical interventions. Both physician and patient discuss possible outcomes and risks and agree on how to proceed [17]. For this model to succeed, it is paramount that physicians are well informed and follow the advice of evidence-based medicine to discuss and clarify patients’ questions based on the most up-to-date evidence [18, 19]. In Switzerland, the Swiss Academy of Medical Sciences [20] and the Swiss Medical Association [21] recommend shared decision-making.

Therefore, in a system where medical decisions are shared, patients’ expectations are a driving force of the appropriate use of the health care system. To design efficient preventive interventions and promote the rational use of the system, it is critical to know patients’ expectations and align them with current evidence-based recommendations. This study aimed to contribute to close this knowledge gap by elucidating the expectations of the adult Swiss general population on check-up prevention.

## Methods

### Ethical statement

Guidance was sought from the Ethics Committee of Northwest and Central Switzerland (EKNZ), which advised that formal ethical approval was not required since the survey complies with the general ethical and scientific standards for research with humans (Project-ID: Req-2019-00896).

Verbal consent was asked before starting each interview. First, interviewers introduced themselves and informed about the survey topic. Then they asked if the person wanted to participate in the interview (with anonymous data collection). Upon verbal acceptance, the survey started. Verbal refusal to participate was coded to do not contact the person again for this study.

### Study design

A nationwide cross-sectional survey conducted in a representative sample of the Swiss adult general population, using computer-assisted telephone interviewing.

### Participants’ selection and data collection

The LINK Institute (https://www.link.ch/), an independent market research company, interviewed a representative sample of the Swiss adult population by telephone, respecting the quotas for sex, age (quotas are based on four age groups: 18 to 29, 30 to 44, 45 to 59, 60 to 79 years old) and language- speaking region (German, French, and Italian) according to the statistics of the Swiss general population. The inclusion criteria for participation were, (i) to be a Swiss resident, (ii) aged 18 years old or older, and (iii) speak German, French or Italian. Exclusion criteria were, (i) inability to follow the questions or give consent or, (ii) insufficient knowledge of one of the study languages. Contacts were generated through Random-Digit-Dialling within the Link Institute’s panel. This method allows for excellent coverage of the Swiss population including persons only reachable by mobile phone or who have an unregistered landline connection. All computer-assisted telephone interviews were conducted by native speakers in Switzerland (in German, French or Italian). Interviewers were specifically trained for this study and continuous supervision took place during the interviews to guarantee high survey quality allowing to resolve any queries at any time.

### Survey

The survey followed a fully structured questionnaire with semi-open and closed questions and lasted about 10 to 12 minutes to complete. After potential participants were informed about the study goal and asked for consent for participation, data were anonymously collected. Participants were asked about their expectations to undergo check-up examinations in addition to regular care at their age. The participants who responded “Yes” to this question (expected check-ups in addition to regular care), were then asked which medical tests and procedures they would expect to be part of such an examination, how often it should take place, and at which age a check-up routine should start. The questions about medical procedures were randomised between blocks (blocks were counselling procedures, sex-specific procedures, and diagnostic tests) to avoid response bias. All participants were asked if they expected their physicians to offer preventive procedures during regular consultations, known as opportunistic prevention (for example, a patient consulting for a cold and then being offered by the physician to check on the vaccination status). Participants responding “Yes” to this question and who had previously reported that that they did not expect check-up examinations were then also asked about medical tests and procedures as previously described. All participants also gave their opinion on their previous knowledge about check-up examinations recommendations, the importance they attributed to having their health status regularly checked, and their subjective health status (between 0, very unhealthy, to 100, very healthy). Survey questions can be found in the S1 Survey.

### Sample size and demographic weighting

We aimed for 1000 completed interviews. With this number the maximum range of variation lies within +/-3.2%, which enables for meaningful analysis between different socio-demographic groups. The Link Institute applied weighting according to the representative distribution of age sex, and language region in Switzerland [22]. Therefore, conclusions for the whole population can be made from the sample.

### Analyses

Analyses were done with the R software [23] and mainly with the package “survey” [24]. All results presented were computed on the weighted data. Predictor variables of check-up expectations were organized in different groups for the analysis. 1) Demographic variables: sex, age group (18 to 29, 30 to 44, 45 to 59, 60 to 79 years old), language region (German, French, Italian), area type (urban or rural), civil status (married, single, concubinage, divorced, widow), employment situation (employed full time (more than 90%), employed part-time (between 50 and 89%), employed part-time (less than 50%), non-employed), and education (primary school, secondary school, professional school, middle school, technical school, university). 2) Relationship to general practitioner (GP) variables: being registered with a GP, the sex of the GP, last visit to the GP, and last check-up examination. 3) General opinions about health checks which included: “did you hear about check-ups?”, “do you have an opinion about check-ups?”, “do you think check-ups are generally necessary?”, “do you think check-ups are generally recommended?”. To test for significant associations between check-up expectations and the above-mentioned predictor variables, we used the function “svychisq” from the R package “survey” and Fishers exact test when necessary. Three generalized linear models with main effects (without interactions) were computed to predict check-up expectations (binary responses of “Yes” and “No") by demographic variables (model 1), relationship to GP (model 2), and general opinions (model 3) (“svyglm” function from “survey” package). Within each model, variables were checked for multicollinearity computing the variance inflation factor (with “vif” function from “car” package [25]), and variables exceeding a value of 5 were excluded [26]. To further assess multicollinearity effects we computed bivariate correlations among all predictors using the function “hetcor" from the R package “polycor” [27]. Highly correlated predictors were removed, namely “civil status” which was highly correlated with “age group” and “thinking that check-ups are necessary" which was highly correlated with “thinking that check-ups are recommended”. Models were recomputed without these variables.

A fourth and more complete generalized linear model was build including all predictors of the former three models and multicollinearity was once again checked using vif computation. Model fit was assessed with McFadden Pseudo R^2^ (“sum” function from “jtools” package [28] where values between 0.2 and 0.4 are considered a very good fit [29]. Subgroup analyses were computed for the respondents who expected check-ups and those who did not expect check-ups. For each group, the expectations for each medical intervention from a list (19 medical interventions independent of sex, plus one specific intervention for men and three specific interventions for women) were calculated and reported as proportions. For the group who expected check-ups, we additionally calculated the frequency of check-up interventions (every 6 months, each year, every two years, every five years, once, or other) and the age at which they were expected. Similarly, the frequency of medical interventions expectations was calculated based on age group.

Further, the age of initial expected check-up routing between men and women was compared using a two-sample t-test using “svyttest”. We also pooled the expectations to each intervention of all participants (including those who expected only check-ups, those who expected only opportunistic prevention, and those who expected both). For each age group, we indicated the percentage of people who expected each intervention at their own age, and in total. We combined this information with the current Swiss guideline recommendations.

## Results

**Fig 1:**
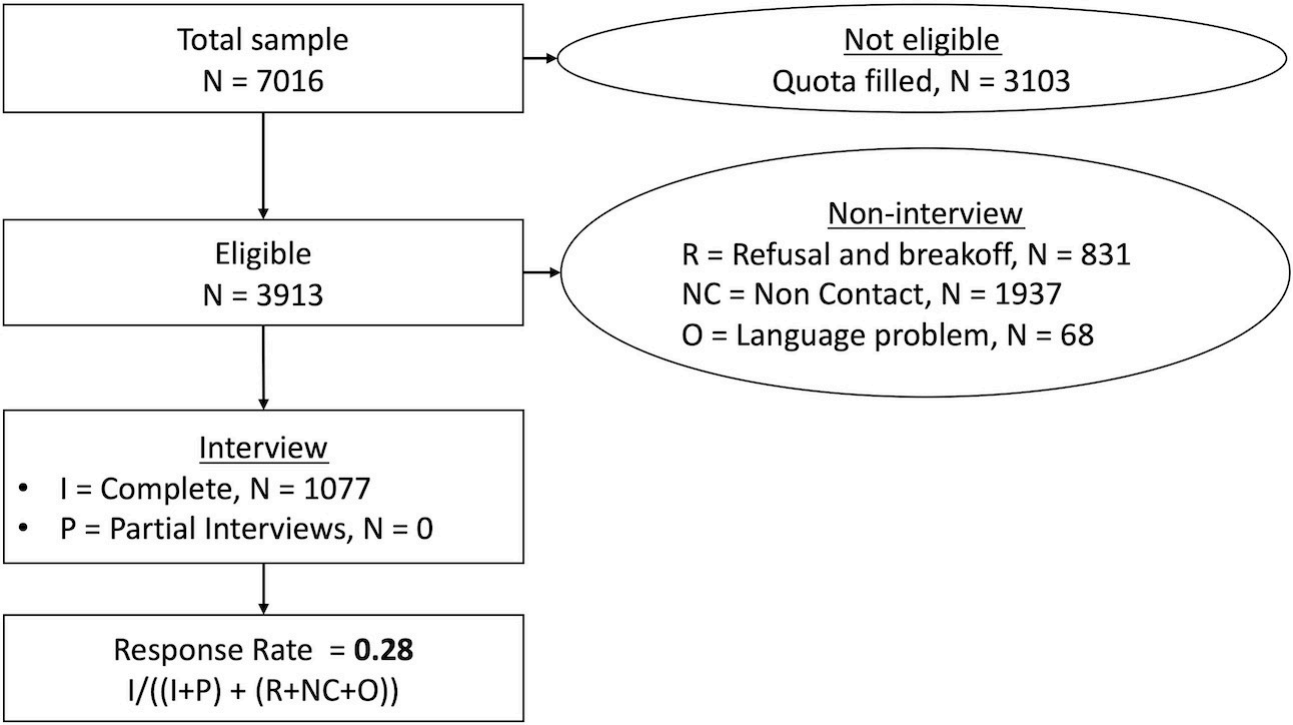
Study recruitment flowchart and response rate.

Initially, 7,016 telephone numbers were randomly generated, of which 3103 had to be discarded given quotas overflow, leaving 3913 eligible numbers. Then, 2836 people were not interviewed because of refusal to participate, inability to reach the contact, or language problems. Finally, 1077 people were interviewed. The response rate was 28% (see survey recruitment flow in Fig 1).

Of the 1077 interviewees, 51% were female, 71% from the German-speaking, 24% from the French-speaking and 6% from the Italian-speaking regions. Mean age was 45 years (range 18 to 89 years old). A description of the Swiss population can be found in S3 Table. Overall, 90% were registered with a GP, 61% had their last GP consultation within the last 12 months, and 41% declared having had a preventive check-up examination during the last year. Table 1 shows respondents general demographic characteristics, and their expectations of check-up examinations and opportunistic prevention.

**Table 1:** Frequency of expectations of check-ups and opportunistic prevention across general demographic characteristics.

	Expects check-ups				Expects opportunistic prevention			
	Total n (%)	Yes n (%)	No n (%)	stats	Total n (%)	Yes n (%)	No n (%)	stats
	1035	429 (41%)	606 (59%)		1045	456 (44%)	589 (56%)	
**Sex**								
Male	525 (51%)	232 (44%)	293 (56%)	Chi = 3.07	530 (51%)	254 (48%)	276 (52%)	Chi = 7.48
Female	510 (49%)	197 (39%)	313 (61%)	*P* = 0.08	516 (49%)	203 (39%)	313 (61%)	*P* = 0.006
**Age group**								
18 to 29 years old	218 (21%)	63 (29%)	155 (71%)	Chi = 31.74	220 (21%)	111 (50%)	109 (50%)	Chi = 15.35
30 to 44 years old	303 (29%)	116 (38%)	187 (62%)	*P*<0.001	305 (29%)	132 (43%)	173 (57%)	*P*<0.001
45 to 59 years old	309 (30%)	163 (53%)	146 (47%)		317 (30%)	147 (46%)	170 (54%)	
60 to 79 years old	205 (20%)	87 (42%)	118 (58%)		203 (19%)	66 (33%)	137 (67%)	
**Language region**								
German	741 (72%)	316 (43%)	425 (57%)	Chi = 6.42	742 (71%)	321 (43%)	421 (57%)	Chi = 7.42
French	236 (23%)	83 (35%)	153 (65%)	*P* = 0.04	245 (23%)	100 (41%)	145 (59%)	*P* = 0.025
Italian	59 (6%)	30 (51%)	29 (49%)		58 (6%)	35 (60%)	23 (40%)	
**Area type**								
Urban	794 (77%)	333 (42%)	461 (58%)	Chi = 0.26	802 (77%)	353 (44%)	449 (56%)	Chi = 0.14
Rural	241 (23%)	96 (40%)	145 (60%)	*P* = 0.613	243 (23%)	103 (42%)	140 (58%)	*P* = 0.708
**Civil status**								
Single	357 (35%)	127 (36%)	230 (64%)	Chi = 12.51	361 (35%)	171 (47%)	190 (53%)	Chi = 6.42
Married	492 (48%)	219 (45%)	273 (55%)	*P* = 0.014	501 (48%)	211 (42%)	290 (58%)	*P* = 0.17
Concubinage	23 (2%)	15 (65%)	8 (35%)		24 (2%)	13 (54%)	11 (46%)	
Divorced	124 (12%)	53 (43%)	71 (57%)		117 (11%)	47 (40%)	70 (60%)	
Widow	36 (3%)	14 (39%)	22 (61%)		38 (4%)	12 (32%)	26 (68%)	
**Employment**								
Full time (90% or more)	475 (46%)	207 (44%)	268 (56%)	Chi = 3.85	483 (46%)	230 (48%)	253 (52%)	Chi = 7.24
Part time (50 to 89%)	242 (23%)	99 (41%)	143 (59%)	*P* = 0.278	242 (23%)	100 (41%)	142 (59%)	*P* = 0.065
Part time (less than 50%)	81 (8%)	26 (32%)	55 (68%)		83 (8%)	37 (45%)	46 (55%)	
Not working	239 (23%)	98 (41%)	141 (59%)		237 (23%)	89 (38%)	148 (62%)	
**Education**								
Primary School	56 (5%)	26 (46%)	30 (54%)	Chi = 6.99	56 (5%)	26 (46%)	30 (54%)	Chi = 2.33
Secondary School	302 (29%)	116 (38%)	186 (62%)	*P* = 0.222	300 (29%)	124 (41%)	176 (59%)	*P* = 0.802
Professional School	242 (24%)	107 (44%)	135 (56%)		247 (24%)	108 (44%)	139 (56%)	
Middle School	74 (7%)	35 (47%)	39 (53%)		74 (7%)	35 (47%)	39 (53%)	
Technical School	154 (15%)	53 (34%)	101 (66%)		159 (15%)	64 (40%)	95 (60%)	
University	197 (19%)	89 (45%)	108 (55%)		198 (19%)	93 (47%)	105 (53%)	

The table presents total numbers (and corresponding percentage in parenthesis) of expectations of check-ups and opportunistic prevention across demographic categories. Results are based on weighted data and are rounded; therefore, totals might not add up in each case. Possible answers to each question where “Yes”, “No”, and “I don’t know”. Only responses “Yes" and "No" are presented here (“I don’t know” responses had less than 8 cases per category).

Chi = Chi-square test

*P* = *P* value

The survey asked general opinions about health check-up examinations. Overall, more than half of respondents had already heard about check-up examinations and had an opinion. They also thought that check-ups are generally necessary and recommended. Regarding the importance attributed to have one’s health regularly checked, 40% consider it “Important” and 23% “Very important” (Table 2).

**Table 2:** Opinion variables descriptives in proportion (n = 1077).

Question	Yes	95% CI
*Have you heard about check-up examinations*?	83%	(81–85%)
*Did you think about this and have an opinion*?	67%	(64–70%)
*Do you think check-ups are generally necessary*?	57%	(54–60%)
*Do you think check-ups are generally recommended*?	68%	(66–71%)
*How important is for you to have your health regularly checked*?		
Not at all important	8%	(6–10%)
Not so important	27%	(25–30%)
Important	41%	(38–44%)
Very important	23%	(21–26%)

CI = Confidence Interval

**Fig 2:**
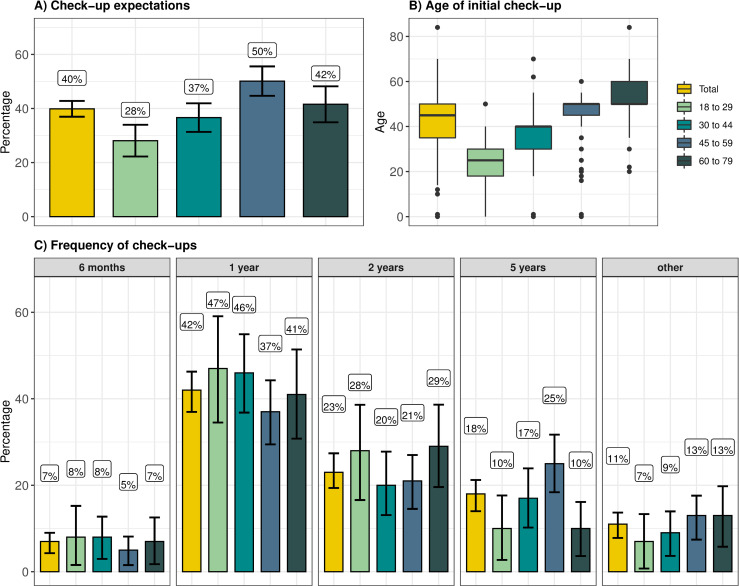
Check-up expectations per age groups and total. A) Percentage of respondents who indicated they expected check-up examinations in addition to regular care per age and in total. B) Age of initial check-up routine expected in each age group and in total. C) Frequency of expected check-ups per age group and in total. Response options were every 6 months, each year, every two years, every five years, once, or other. No respondent chose the option ‘once’, therefore this is not shown in the figure.

The survey followed asking if the person expected to have check-ups at their age. From the entire sample, 40% (n = 429) of respondents indicated that they expected to have check-up examinations in addition to regular care, while 56% did not expect so, and 4% responded: “I don’t know”. Fig 2A shows the positive expectation of check-ups, for the whole sample and each age group. From those who expected to have check-ups, 42% expected to have the examinations yearly, 23% every two years, 18% every five years, and 7% every six months. The response option “other” was chosen by 11% of respondents. It was also possible to respond “once in a lifetime” but no one chose it, and it is therefore not represented in Fig 2C. The age most often indicated to start having check-ups was 50 years old (chosen by 35% of respondents, median = 45 years old). As shown in Fig 2B respondents from each age group expected to start the check-up routine around their age. Among those aged 18 to 29 years, the age chosen most often (by 23% of respondents in this age group) was 22 years old (median = 25); among those between 30 and 44, 27% expected to start at 40 years old (median = 40); among those between 45 and 59, 57% expected to start at 50 years old (median = 50), and among those between 60 and 79, 47% expected to start the check-up routine at 50 years old (median = 50). There were no statistical differences between men and women regarding the age at which to start the check-up routine (t.test = -1.7921, *P* value = 0.07).

All respondents were also asked if they expected to have preventive procedures offered during a regular consultation, known as opportunistic prevention. Overall, 42% said “Yes”, while 55% said “No” and 3% responded “I don’t know”, (see Table 1 for demographic characteristics of the respondents). Table 3 shows the proportions of respondents expecting opportunistic prevention based on their expectation of check-up examinations. Notably, 61% of those who expected to have check-up examinations, also expected to have opportunistic prevention.

**Table 3:** Relationship between expectation of check-ups and expectation of opportunistic prevention.

	Expects opportunistic prevention			
	Yes (n = 456)	No + I don’t know (n = 621)		
**Expects Check-up Yes** (n = 429)	(n = 261) 61%	(n = 168) 39%	Chi	98.7
**Expects Check-up No + I don’t know** (n = 647)	(n = 195) 30%	(n = 453) 70%	*P* value	<0.001

Chi = Chi square test

### Medical interventions expectations

**Fig 3:**
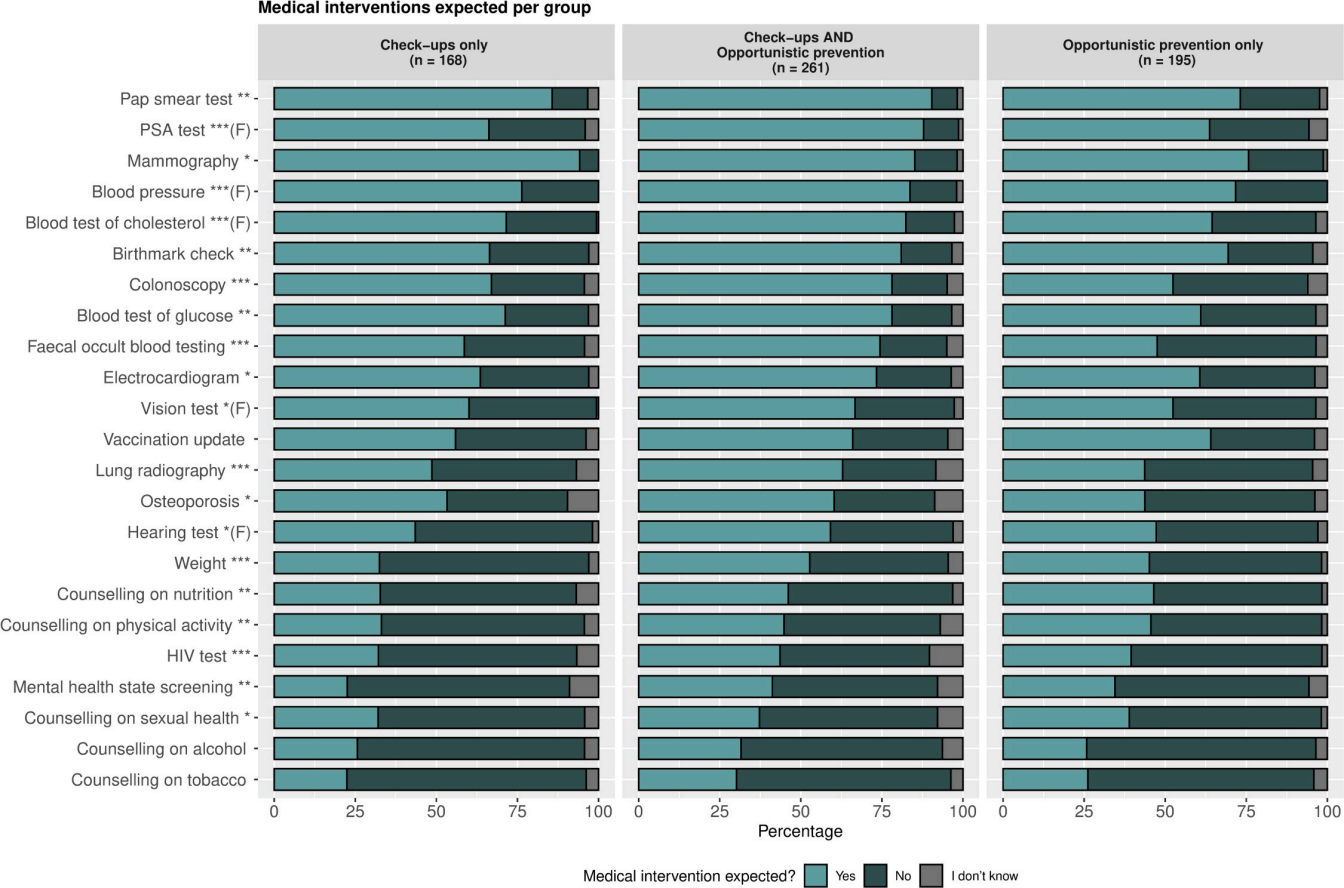
Medical interventions per group. The expectation for each medical intervention for participants expecting only check-ups, participants expecting check-ups and opportunistic prevention, and participants expecting only opportunistic prevention. Significant differences per each medical intervention were calculated with Chi-square t, or Fishers exact test when necessary (marked with (F) next to the medical intervention). Pap smear test = Papanicolau smear test for cervical cancer screening. PSA test = Prostate Specific Antigen blood test. * *P* value < 0.05, ** *P* value < 0.01, *** *P* value < 0.001.

Regarding the medical interventions expected to be part of prevention, participants rated each intervention from a list as shown in Fig 3. The most highly expected tests were those sex-specific, like mammography (89% of women), Pap smear test (89% of women), and blood test of prostate-specific antigen (PSA) (81% of men). Followed by other common procedures like blood pressure (81%), blood test of cholesterol and lipid disorders (78%), blood test of glucose (76%), and birthmarks check for skin cancer prevention (75%). The least favoured interventions were those of counselling and mental health; as counselling on tobacco (27%), counselling on alcohol abuse (29%), and mental health state screening (35%).

### Expected interventions per groups

We subdivided the sample into three groups based on the type of prevention they expected. (1) Those expecting only check-ups, (2) those expecting check-ups and opportunistic prevention, and (3) those expecting only opportunistic prevention. In general, the group expecting check-ups and opportunistic prevention was the group expecting more medical interventions as can be seen in Fig 3. Regarding the frequency in which these interventions should take place, most respondents expected them each ear except for vaccination updates, hearing test, and colonoscopy deemed necessary every five years.

The expectations for specific medical interventions were different among age groups. Younger participants expected more often all counselling interventions, mental health state/depression screening, HIV testing, skin cancer prevention, vaccination update, low dose CT lungs scan, and weight/obesity assessment than older participants. There were no differences regarding the expectations of the sex-specific interventions (Pap smear test, mammography, osteoporosis, and PSA test) among the different age groups. Neither were there age differences in the expectation of blood pressure, blood test of cholesterol and other lipid disorders, faecal occult blood testing, electrocardiogram, and hearing test. Colonoscopy was more expected in the older age groups than in the younger ones (See S1 Fig and accompanying table in the supplementary materials for further detail).

### Predictors of check-up expectations

The first model assessed demographic variables as predictors of check-up expectations. Significant predictors of check-up expectations were age group, where older respondents were more likely to expect check-ups than the younger (‘30 to 44 years old’ OR = 1.51, 95% CI: 1.01–2.26, *P* value = 0.046, ‘45 to 59 years old’, OR = 2.90, 95% CI: 1.95–4.31, *P* value <0.001, ‘60 to 79 years old’ OR = 1.88, 95 CI: 1.18–3.01, *P* value = 0.008), language region, where participants from the French speaking region were less likely to expect check-ups than their counterparts from the German speaking region (OR = 0.69, 95% CI: 0.49–0.96, *P* value = 0.029), employment status, where respondents employed less than 50% were less likely to expect check-ups than those employed full time (OR = 0.58, 95% CI: 0.34–0.99, *P* value = 0.048), and finally education, where those with a University degree where more likely to expect check-ups than those holding a Secondary school degree (OR = 1.5, 95% CIs: 1.03–2.2, *P* value = 0.036, S1A Table).

The second model assessed GP-related variables as predictors of check-up expectations. Significant predictors were having had the last check-up examination 3 years ago or longer (OR = 0.44, 95% CI: 0.28–0.71, *P* = 0.001) or never having had a check-up examination (OR = 0.23, 95% CI: 0.16–0.33, *P* < 0.001, compared with having had the last check-up examination within the last six months, S1B Table).

The third model assessed general opinions about check-ups as predictors of check-up expectations. We found that respondents who did not have an opinion about check-ups, or responded, “I don’t know”, were less likely to expect them than those who reported having a formed opinion (‘No’: OR = 0.52, 95% CI: 0.34–0.81, *P* = 0.00 and ‘I don’t know’: OR = 0.39, 95% CI: 0.26–0.6, *P* < 0.001). Likewise, those respondents thinking that check-ups are not recommended or did not know about the recommendation were less likely to expect them than respondents thinking that check-ups are recommended (‘No’: OR = 0.27, 95% CI: 0.18–0.4, *P* < 0.001 and ‘I don’t know’: OR = 0.46, 95% CIs: 0.25–0.86, *P* = 0.015). Finally, respondents who attributed more importance to regularly checking one’s health were more likely to expect check-ups than those attributing less importance (OR = 2.26, 95% CIs: 1.87–2.74, *P* < 0.001) (S1C Table).

The fourth and most complete model controlled for all three sets of predictors and the subjective health status, as shown in Table 4. Significant predictors for expecting check-ups were being male, age between 45 and 59 years old (compared to 18 to 29 years olds), having a degree from professional, middle school or university (compared to having a degree from secondary school), and the more importance attributed to having one’s health regularly checked. Significant predictors for not expecting check-ups were, to be employed less than 50% (compared to full time), to never have had a check-up (compared to having had one in the last 6 months), to do not have an opinion about check-ups, or do not know (compared to having an opinion) and finally thinking that check-ups are not recommended or do not know about the recommendation (compared to thinking that check-ups are recommended). This model was the best fitting one given McFadden Pseudo R^2^.

**Table 4:** Factors associated with check-up expectations.

Factors associated with the expectation of having check-up examination. Multivariable-adjusted logistical regression (N = 987 observations). Model 4 including demographic, GP related and opinion variables.				
	Expects to have check-up examinations			
Variables	OR	95% CI	*P* value	Overall *P* value
**Sex**				
Female (Reference)	1			
Male	1.45	(1.02–2.05)	**0.040**	
**Age group**				
18 to 29 years old (reference)	1			
30 to 44 years old	1.32	(0.83–2.11)	0.236	0.018
45 to 59 years old	2.03	(1.27–3.23)	**0.003**	
60 to 79 years old	1.31	(0.77–2.22)	0.323	
**Language region**				
German (reference)	1			
French	0.76	(0.5–1.16)	0.198	0.443
Italian	0.90	(0.44–1.85)	0.770	
**Area type**				
Urban (reference)	1			
Rural	0.87	(0.61–1.26)	0.464	
**Employment**				
Full time (90% or more) (reference)	1			
Part time (50 to 89%)	0.85	(0.55–1.31)	0.474	0.167
Part time (less than 50%)	0.55	(0.31–0.99)	**0.045**	
Not working	0.67	(0.41–1.09)	0.104	
**Education**				
Secondary School (reference)	1			
Primary School	1.61	(0.8–3.24)	0.180	**0.022**
Professional School	1.73	(1.11–2.69)	**0.015**	
Middle School	1.99	(1.04–3.78)	**0.037**	
Technical School	0.96	(0.59–1.55)	0.857	
University	1.66	(1.06–2.61)	**0.026**	
**Last check-up examination**				
Within the last 6 months (reference)	1			
1 year ago	0.82	(0.54–1.25)	0.359	**0.007**
2 years ago	0.93	(0.53–1.64)	0.801	
3 years ago	0.74	(0.32–1.72)	0.489	
More than 3 years ago	0.74	(0.43–1.28)	0.282	
Never had a check-up	0.41	(0.26–0.65)	**<0.001**	
**Last GP visit**				
Less than 12 months ago (refence)	1			
12 to 24 months ago	0.94	(0.61–1.46)	0.798	0.383
More 24 months ago	1.42	(0.84–2.4)	0.186	
I don’t know	0.79	(0.45–1.39)	0.416	
**Do you have an opinion about check-ups**				
Yes (refence)	1			
No	0.62	(0.38–1)	**0.049**	**0.014**
I don’t know	0.54	(0.33–0.88)	**0.013**	
**Importance of regularly checking one’s health (per 1 point)**	2.12	(1.71–2.63)	**<0.001**	
**Do you think check-ups are recommended**				
Yes (reference)	1			
No	0.26	(0.17–0.4)	**<0.001**	**< 0.001**
I don’t know	0.46	(0.23–0.92)	**0.028**	** **
**Perceived health status** (per 10 points)	0.96	(0.87–1.05)	0.344	

Multivariable model for the binary response "Yes, expects check-ups "vs "No, does not expect check-ups", adjusted for sex, age group, language region, area type, employment, education, date to last check-up examination, date to last GP visit, having an opinion about check-ups, importance of regularly checking one’s health status, thinking check-ups are recommended and subjective perceived health status (between 0, feeling very unhealthy, and 100, feeling very healthy).

McFadden Pseudo R squared = 0.2213

OR: Odds Ration, CIs: Confidence Interval

Respondents also rated their subjective health status at the moment of the interview between 0 (feeling very unhealthy) to 100 (feeling very healthy). The mean subjective health state differed slightly between the groups of check-up expectations. Respondents who expected to have check-ups scored on average 83, while those who did not expect check-ups scored on average at 85, and finally respondents who did not know about their expectations on check-ups scored lower at 76. Those differences were not significant when the variable was introduced in the complete regression model, as seen in Table 3. (Also, see S1D Table for the unadjusted results).

### Recommendations and general expectations

We further pooled together the expectations for each preventive intervention from all participants, (including those expecting check-ups and those expecting opportunistic prevention) and combined them in a table with the corresponding Swiss guidelines. Table 5 presents the results for the overall sample and S2 Table details the results per age group. Overall participants expected counselling interventions between 28% (tobacco) and 44% (nutrition and physical activity). They expected cancer prevention more often between 58% (low CT scan for lung cancer) and 79% (blood test of PSA). Vaccination update was expected 65% overall. Regarding other screening procedures, expectations varied between 38% for psychological state/depression assessment to 79% for blood pressure control.

**Table 5:** Percentage of expected interventions and guideline recommendations.

Intervention Swiss guideline recommendation (EviPrev)	n—expects the test	%	lower 95% CI	upper 95% CI
**Counselling on tobacco (n = 579)**	163	28	24.48	31.81
Recommended to all ages				
**Counseling on alcohol (n = 574)**	171	30	26.11	33.60
Recommended to all ages				
**Counselling on nutrition (n = 577)**	257	44	40.43	48.54
If BMC > 27 kg/m2 + CV RF Recommended with counselling on physical activity.				
**Counselling on physical activity (n = 575)**	252	44	39.84	47.96
If BMC > 27 kg/m2 + CV RF Recommended with counselling on nutrition.				
**Counselling on sexual health (n = 573)**	217	38	33.95	41.90
Recommended to the population at risk				
**low dose CT scan for lung cancer (n = 563)**	324	58	53.47	61.64
Between 55 and 80 y. (> 30 UPA, smoker or stopped smoking < 15 years ago)				
**Skin cancer prevention (n = 582)**	445	76	73.03	79.93
Not recommended				
**Colonoscopy (n = 573)**	409	71	67.61	75.02
From 50 y. > every 10 years				
**Faecal occult blood testing (n = 576)**	377	66	61.68	69.44
From 50 y. > every 2 years.				
**Blood test of PSA (n = 313) [M]**	246	79	74.01	83.10
Not recommended				
**Mammography (n = 276) [W]**	237	86	82.04	90.21
From 50 y. > every 2 years.				
**Papsmear test / cytology (n = 273) [W]**	235	86	82.01	90.22
Between 25 and 65 y. > every 3 years				
**Vaccination update (n = 578)**	378	65	61.53	69.29
Recommended to all ages				
**HIV screening (n = 562)**	237	42	38.11	46.28
Recommended to the population at risk				
**Depression (n = 560)**	211	38	33.67	41.70
Recommended to all ages				
**Blood test of lipid disorders (n = 589)**	448	76	72.61	79.50
[M]> 35 y. and [W] > 45y. > every 5 years With CV RF: individual				
**Blood test of glucose (n = 583)**	432	74	70.57	77.68
From 40 y. With RF or BMI > 25 kg/m2: individual				
**Blood pressure (n = 599)**	471	79	75.36	81.92
Recommended to all ages				
**Weight/obesity (n = 582)**	272	47	42.66	50.76
Recommended to all ages				
**Osteoporosis (n = 257) [W]**	149	58	51.86	63.93
With RF: from 50 y. Without RF: From 65 y.				
**Vision test (n = 590)**	370	63	58.77	66.58
Not recommended				
**Hearing test (n = 588)**	314	53	49.31	57.37
Not recommended				
**Electrocardiogram (n = 583)**	406	70	65.97	73.43
Not recommended				

Table shows the expectations for each medical intervention from all survey participants who either expected to have check-ups in addition to regular care and/or opportunistic prevention.

Number in parenthesis next to the intervention title is the number of people who responded yes/no to the expectation of the intervention. Otherwise specified the intervention is meant for both men and women. If not otherwise specified, recommendations stop at 75 years old.

Legend: eviprev = https://eviprev.ch/wp-content/uploads/2019/11/tableau_oct2016_f.pdf, CT = computed tomography, CV = cardiovascular, RF = risk factors, UPA = Unit package year y. = years old, [M] = only men, [W] = only women

## Discussion

We investigated the general population’s expectations regarding preventive interventions, namely check-up examinations in asymptomatic adults and opportunistic prevention during regular care visits with the physician. Most interviewees had heard beforehand about check-up examinations and had an opinion about it. Precisely, 40% expected to have check-up examinations at their age in addition to regular care. Per age groups, 28% of those aged 18 to 29 years old expected check-ups, while this proportion increased with age to 37% for those aged 30 to 44 years old, with a maximum of 50% of those aged 45 to 59 years old. Check-up expectations decreased to 42% for those aged 60 and above. This pattern is according to the literature regarding differences in health-seeking behaviour with age [30]. In Switzerland, general check-up examinations in asymptomatic adults are generally not recommended. Guidelines recommend performing check-up/case-finding interventions according to age, sex, and individualized patient risk [8].

Participants indicated their expectations of preventive medical interventions. There was a strong interest in sex-specific interventions (Pap smear, PSA test, mammography), followed by blood pressure and blood controls (lipids, glucose), different types of cancer screening, and vaccination updates. Expectations decreased for counselling on lifestyle factors, namely advice on tobacco, alcohol abuse, nutrition, physical activity, and sexual behaviour. The overestimation of the expected check-up tests follows the pattern shown by previous studies. Sa et al., reported that the Portuguese population overrate prevention practices and favoured laboratory testing over lifestyle counselling [14]. Martins et al. showed the general belief of the need to use a lot of services on a nearly annual basis, which would cause the overuse of resources [15].

It is worth noting that 42% of the sample did not expect check-ups nor opportunistic prevention. These results point in the direction of other studies, showing that when patients are given the opportunity, only about a third can raise questions related to prevention or know which preventive interventions would be relevant from them and when [31, 32].

### Counselling expectations and recommendations

Overall, 28% and 30% of respondents expected tobacco and alcohol abuse counselling respectively, while these guideline recommendations recommend these interventions to all ages. People expected counselling on nutrition and physical exercise 44% on average, and these interventions are recommended to be combined in patients with a BMI above 27 kg/m^2^ and with cardiovascular risk. Counselling on sexual health was expected by 38% of respondents, with a peak of interest in the youngest age group (68%) and a progressive decrease with age (28% for the oldest). This intervention is nevertheless recommended to the population at risk independent of age.

Expectation prevalence for lifestyle counselling is partially following how much counselling is usually delivered in practice. In general, nutrition counselling is provided at around 35% of regular visits, while physical exercise advice around 26% of visits or less [33–36]. A possible reason why patients would not expect weight and nutrition counselling from their GPs could be that some people believe that dieticians are most qualified to give this advice. Practice nurses and physicians are ranked fourth for this kind of counselling [37]. In our study, counselling on tobacco was the least expected intervention overall. This expectation follows the pattern shown by other studies where even when 60% of patients were screened for tobacco use, among those classified as current tobacco users, only 20.9% received tobacco counselling during their visit with a physician [38]. Sa et al., also reported that the general Portuguese population attributed the least importance to counselling on tobacco and alcohol abuse. The minimal interest in counselling follows the Swiss study of Cornuz et al., where they linked subjective physicians’ barriers in several prevention interventions to patient’s lack of interest among others [39]. Also, others reported that patients indicated that primary preventive care was only appropriate when associated with concrete actions or tests, such as cholesterol tests and Pap smears [31].

Counselling is paramount to promote lifestyle behaviour change. It has been shown that when physicians do not discuss it or at least mention it, patients understand that they should continue their current behaviours. Therefore, omitting to address lifestyle changes also influences the behaviour of patients [40], and the failure to counsel translates into missed opportunities for primary prevention [36].

### Cancer screening expectations and recommendations

Respondents were generally very interested in cancer screening interventions. Especially expected were the sex-specific procedures. For example, 86% if women overall expected a Pap smear test, and it is recommended to all women between 25 and 65 years of age (every three years). 86% of women expected a mammography, although it is recommended only from 50 years old every two years. Notably, there were no significant differences in expectations per age group in these two interventions. Overall, 79% of men expected a blood test of PSA. Interest increased with age, although this procedure is currently not recommended to any age group. The now outdated recommendation to regularly screen PSA might drive the high expectation. Evidence shows that PSA test screening entails a very small reduction in mortality, but has risks of overdiagnosis, overtreatment, false-positive results, and harms associated with screening and diagnostic. The USPSTF concludes that the benefits of PSA test screening for prostate cancer might not outweigh the harms [41]. Colonoscopy expectations increased with age (from 54% the youngest to 80% the oldest age group), partially corresponding with the recommendation to start this check at 50 years old and repeat it every ten years. Feacal occult blood testing was slightly less expected than colonoscopy but followed the same pattern with increasing age to a maximum of 74% for people aged 60 and older. Guidelines recommend this procedure from 50 years old every two years. A low dose CT scan to screen for lung cancer was overall expected by 58%, with the younger showing more interest. Guidelines only recommend this screening for smokers (>30 UPA) and ex-smokers (stopped smoking <15 years ago) from 55 to 80 years. Screening for skin cancer was overall expected by 76%, with the highest interest in the younger age group (84%) and decreased with increasing age (older group 69%). Contrary, guidelines do not specifically recommend this screening as there is not enough evidence to recommend it or not in asymptomatic adults [42].

### Other screening procedures expectations and recommendations

Vaccination update expectation was highest for the younger group and slightly decreased with age, with an overall interest of 65%. Guidelines stress the benefit of checking the vaccination status and vaccinate according to risk and age. As expected, and probably reflecting risk behaviour, younger respondents were also the most interested in HIV testing, with 71% expectation, while the older population group had very little expectation, at 22%. Guidelines recommend HIV testing to anyone at risk independently of age. Regarding mental health status/depression screening, there was an overall expectation of 38% (ranging from 57% for the youngest to 25% for the oldest age groups). Nevertheless, there is an acceptable degree of evidence for screening for depressive symptoms at all ages. A blood test of cholesterol and lipid disorders (recommended for men over 35 years and women over 40, every five years) was expected overall 76%, with no significant differences among age groups. A blood test of glucose (recommended from 40 years old) was also highly expected overall (74%), and blood pressure testing was expected by 79% and recommended to all ages. It is interesting to compare the percentage of overall expectations for the previous interventions, with other procedures included in the survey but not recommended. For example, vision and hearing testing were overall expected by 63% and 53% respectively, well above the expectations of lifestyle counselling for instance, while none is recommended. Also, the expectation of electrocardiogram by 70%. These results reflect the need for education on preventive practices with good evidence for their cost-effectiveness.

### The cost of health care for the Swiss population

Basic health insurance in Switzerland is mandatory and regulated by the Swiss Federal Law on Compulsory Health Care (LAMal). Each citizen is obliged to have basic private insurance with a franchise ranging between 300 and 2500 CHF per year (for adults). Also, they must pay 10% of each intervention until 700 CHF per year. Additionally, they can apply for additional insurances to cover medical costs not already covered by the basic insurance. In this model, prevention practices are not free of charge and are partially covered by the basic insurance. Therefore, patients pay per procedure according to their franchise and the co-payment deductible as for other interventions. We can hypothesize that based on each person’s monthly insurance costs (premium, franchise, and co-payment) and general financial situation, some people could be more sensitive to charges and decide to undergo or not preventive practices accordingly. Other studies in the US, have shown that the population had a high desire for check-ups but that this was sensitive to charges. We did not ask participants about their health care expenditure, nor their awareness of the costs implied by each intervention for them. We can imagine that expectations for some procedures could slightly change if patients would be aware of the cost, although this argument would be equally valid for non-preventive procedures as usually patients do not ask for the price of an intervention beforehand if they expect it or think they need it (provided they do not leave in the minimum level of subsistence).

### Recommendations to policy makers and physicians

The results of the study highlight the mismatch between guidelines on prevention and the population’s expectations. To achieve a better alignment and consequently better public health we have the following recommendations.

- Counselling is a cost-effective preventive intervention. Given its unpopularity as reported in the literature, the barriers outlined by professionals [39] and, the little expectations for this kind of intervention reported in our study, it would be of interest for physicians and patients alike to see public campaigns addressing the main components included in prevention, namely: counselling, immunization, and physical examination according to the person’s sex, age, and individual risk.
- Regarding the specific preventive medical procedures, physicians need to discuss the benefits and harms of procedures recommended vs. expected and the concept of individualization of risk. On the one side, some interventions would be unnecessary and, on the other hand, if patients are not aware of individual risk, they are less likely to engage in prevention [43].
- Physicians should keep in mind that while certain patients will know and demand interventions they consider necessary, others will not have a clear idea and will need to be guided. The physician needs to lead the conversation on prevention, especially if the patient is not talking about it.

### Limitations

The validity of the study results is based on the correct understanding of the concept ‘check-up examinations while asymptomatic’. We did our best to minimize misunderstandings. We gave a clear definition of the concept at the beginning of the survey and before each relevant question. The Link Institute trained interviewers specifically for this study to emphasize the different nature of a check-up examination versus a regular care consultation. They could clarify the participants in case of doubts at any time during the interview.

The survey was developed in our Institute, and it is therefore not a validated instrument. Nevertheless, the questions were formulated considering other questionnaires previously used in other countries [13–15] and were tested by the authors beforehand among some individuals with and without medical education and then discussed at length with the Link Institute.

We did not specifically ask for individualization of risk knowledge, which does not allow us to compare the survey results with the Swiss guideline recommendations in depth. Neither did we not ask about the participants’ financial situation. Therefore, we cannot rule out that some expectations were driven by it.

## Conclusions

This study elucidated the expectations for check-up examinations of a representative sample of the adult Swiss population. Almost half of the respondents expected to undergo routine health check-ups while being asymptomatic. This expectation conflicts with the current guideline recommendations and therefore challenges the shared decision-making-driven encounter between patient and physician. However, this information is helpful for physicians and policy makers to know that some actions are needed to align expectations with evidence-based recommendations, which in turn would help to have a rational and cost-effective use of the Swiss health care system. The actions suggested are for physicians to discuss the harms and benefits of medical interventions (recommended and not) and increase lifestyle counselling during regular visits. For policy makers, to run public campaigns detailing the main components of prevention, namely, counselling, immunization, and physical examination according to the person’s sex, age, and individual risk.

## Supporting information

S1 Fig Medical interventions expected per age groups. (TIF)

S1 Table Check-up predictors and correlations. A. Demographic variables associated with check-up expectations. B. Relationship to the GP variables associated with check-up expectations. C. Opinion variables associated with check-up expectations. D. Perceived health status association with check-up expectations. S1.E. Predictors correlations.
(PDF)

S2 Table Percentage of expected Interventions per age group and in total. (PDF)

S3 Table Swiss Population 2019. (PDF)

S1 Survey Survey questions in original languages and English. (XLSX)
