# Supplementary figures and images for: Expectations about check-up examinations among Swiss residents: A nationwide population-based cross-sectional survey
Source: PLoS One. 2021 Jul 21;16(7):e0254700. doi: 10.1371/journal.pone.0254700 (PMC8294504; doi:10.1371/journal.pone.0254700)

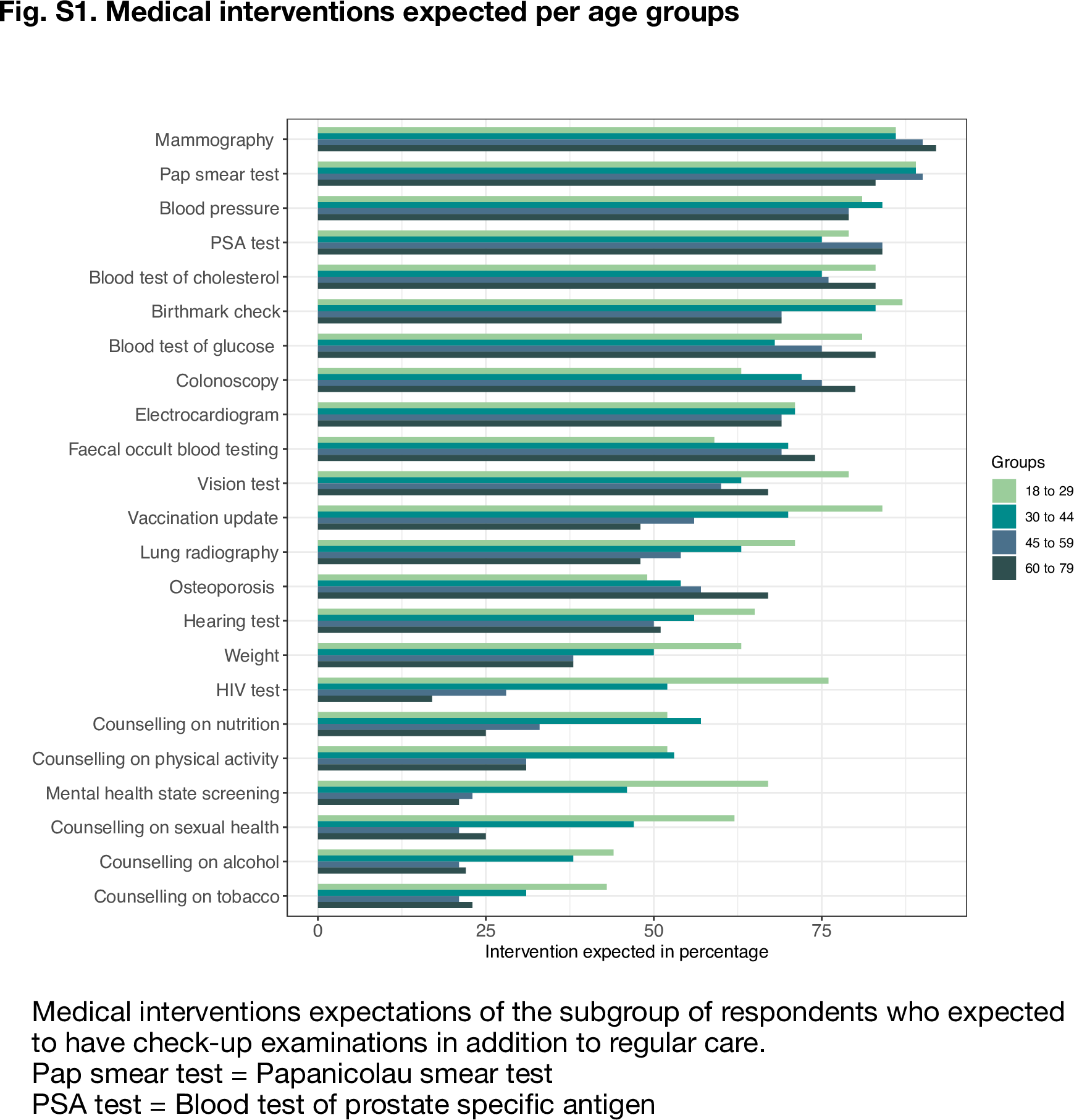

Supplement: S1 Fig — (TIF) [file pone.0254700.s001.tif]
