# Supplementary material for: Expectations about check-up examinations among Swiss residents: A nationwide population-based cross-sectional survey
Source: PLoS One. 2021 Jul 21;16(7):e0254700. doi: 10.1371/journal.pone.0254700 (PMC8294504; doi:10.1371/journal.pone.0254700)
Supplement: S1 Table — A. Demographic variables associated with check-up expectations. B. Relationship to the GP variables associated with check-up expectations. C. Opinion variables associated with check-up expectations. D. Perceived health status association with check-up expectations. S1.E. Predictors correlations. (PDF) [file pone.0254700.s002.pdf]

**Table S1. Check-up predictors and correlations**

**Table S1.A. Demographic variables associated with check-up expectations**

Factors associated with the expectation of having check-ups in addition to regular care.
Multivariable-adjusted logistical regression ( N = 1026 observations)
Model 1, including demographic variables as predictors

| | Expects to have check-up examinations | | | | | | |
|---|---|---|---|---|---|---|---|
| | Adjusted results | | | | Unadjusted results | | |
| **Variables** | **OR** | **95% CI** | ***P* value** | **Overall *P* value** | **OR** | **95% CI** | ***P* value** |
| **Sex** | | | | | | | |
| Female (Reference) | 1 | | | | | | |
| Male | 1.19 | ( 0.89 - 1.59 ) | 0.237 | | 1.28 | ( 1 - 1.64 ) | **0.054** |
| **Age group** | | | | | | | |
| 18 to 29 years old (reference) | 1 | | | | | | |
| 30 to 44 years old | 1.51 | ( 1.01 - 2.26 ) | **0.046** | **<0.001** | 1.54 | ( 1.04 - 2.27 ) | **0.030** |
| 45 to 59 years old | 2.90 | ( 1.95 - 4.31 ) | **<0.001** | | 2.76 | ( 1.89 - 4.03 ) | **< 0.001** |
| 60 to 79 years old | 1.88 | ( 1.18 - 3.01 ) | **0.008** | | 1.83 | ( 1.22 - 2.76 ) | **0.004** |
| **Language region** | | | | | | | |
| German (reference) | 1 | | | | | | |
| French | 0.69 | ( 0.49 - 0.96 ) | **0.029** | **0.038** | 0.72 | ( 0.53 - 0.98 ) | **0.037** |
| Italian | 1.28 | ( 0.74 - 2.2 ) | 0.380 | | 1.45 | ( 0.85 - 2.47 ) | 0.168 |
| **Area type** | | | | | | | |
| Urban (reference) | 1 | | | | | | |
| Rural | 0.93 | ( 0.68 - 1.26 ) | 0.627 | | 0.94 | ( 0.7 - 1.26 ) | 0.679 |
| **Employment** | | | | | | | |
| Full time (90% or more) (referen | 1 | | | | | | |
| Part time (50 to 89%) | 0.88 | ( 0.61 - 1.26 ) | 0.485 | 0.268 | 0.91 | ( 0.66 - 1.24 ) | 0.544 |
| Part time (less than 50%) | 0.58 | ( 0.34 - 0.99 ) | **0.048** | | 0.60 | ( 0.36 - 0.99 ) | **0.045** |
| Not working | 0.90 | ( 0.6 - 1.36 ) | 0.620 | | 0.90 | ( 0.65 - 1.23 ) | 0.508 |
| **Education** | | | | | | | |
| Secondary School (reference) | 1 | | | | | | |
| Primary School | 1.30 | ( 0.73 - 2.3 ) | 0.378 | 0.113 | 1.36 | ( 0.77 - 2.41 ) | 0.290 |
| Professional School | 1.32 | ( 0.91 - 1.9 ) | 0.142 | | 1.26 | ( 0.9 - 1.78 ) | 0.182 |
| Middle School | 1.61 | ( 0.93 - 2.8 ) | 0.091 | | 1.40 | ( 0.84 - 2.34 ) | 0.201 |
| Technical School | 0.91 | ( 0.6 - 1.39 ) | 0.666 | | 0.84 | ( 0.56 - 1.26 ) | 0.406 |
| University | 1.50 | ( 1.03 - 2.2 ) | **0.036** | | 1.32 | ( 0.92 - 1.91 ) | 0.132 |

Multivariable model for the binary response "Yes, expects check-ups "vs "No, does not expect check-ups", adjusted for sex, age group, langauge region, area type, employment and education.
McFadden Pseudo R squared = 0.0401
OR: Odds Ration, CIs: Confidence Interval
Right column shows unadjusted univariable restults for the same variables.

**Table S1.B. Relationship to the GP variables associated with check-up expectations**

Factors associated with the expectation of having check-ups in addition to regular care.
Multivariable-adjusted logistical regression ( N = 998 observations)
Model 2, including relation to GP variables as predictors

| | Expects to have check-up examinations | | | | | | |
|---|---|---|---|---|---|---|---|
| | Adjusted results | | | | Unadjusted results | | |
| **Variables** | **OR** | **95% CI** | ***P* value** | **Overall *P* value** | **OR** | **95% CI** | ***P* value** |
| **Last check-up examination** | | | | | | | |
| Within last 6 months (ref) | 1 | | | | | | |
| 1 year ago | 0.77 | ( 0.53 - 1.11 ) | 0.162 | **< 0.001** | 0.74 | ( 0.51 - 1.07 ) | 0.111 |
| 2 years ago | 0.75 | ( 0.46 - 1.21 ) | 0.237 | | 0.72 | ( 0.45 - 1.16 ) | 0.175 |
| 3 years ago | 0.71 | ( 0.37 - 1.37 ) | 0.313 | | 0.70 | ( 0.37 - 1.33 ) | 0.276 |
| More than 3 years ago | 0.44 | ( 0.28 - 0.71 ) | **0.001** | | 0.42 | ( 0.27 - 0.66 ) | **< 0.001** |
| Never had a check-up | 0.23 | ( 0.16 - 0.33 ) | **<0.001** | | 0.22 | ( 0.15 - 0.32 ) | **< 0.001** |
| **Last GP visit** | | | | | | | |
| Less than 12 months (ref) | 1 | | | | | | |
| 12 to 24 months ago | 0.78 | ( 0.53 - 1.13 ) | 0.190 | 0.419 | 0.66 | ( 0.46 - 0.94 ) | **0.021** |
| More 24 months ago | 1.00 | ( 0.64 - 1.56 ) | 0.999 | | 0.74 | ( 0.49 - 1.11 ) | 0.147 |
| I don't know | 0.76 | ( 0.48 - 1.2 ) | 0.240 | | 0.65 | ( 0.42 - 1.01 ) | **0.053** |

Multivariable model for the binary response "Yes, expects check-ups "vs "No, does not expect check-ups", adjusted for the date to the last check-up examination and the last GP visit.
McFadden Pseudo R squared = 0.0609
OR: Odds Ration, CIs: Confidence Interval
Right column shows unadjusted univariable restults for the same variables.

**Table S1.C. Opinion variables associated with check-up expectations**

Factors associated with the expectation of having check-ups in addition to regular care.
Multivariable-adjusted logistical regression ( N = 1036 observations)
Model 3 including opinion variables as predictors

| | Expects to have check-up examinations | | | | | | |
|---|---|---|---|---|---|---|---|
| | Adjusted results | | | | Unadjusted results | | |
| **Variables** | **OR** | **95% CI** | ***P* value** | **Overall *P* value** | **OR** | **95% CI** | ***P* value** |
| **Do you have an opinion about check-ups** | | | | | | | |
| Yes (refence) | 1 | | | | | | |
| No | 0.52 | ( 0.34 - 0.81 ) | **0.003** | **< 0.001** | 0.42 | ( 0.29 - 0.62 ) | **< 0.001** |
| I don't know | 0.39 | ( 0.26 - 0.6 ) | **<0.001** | | 0.32 | ( 0.22 - 0.46 ) | **< 0.001** |
| **Importance of regularly checking one's health (per 1 point)** | 2.26 | ( 1.87 - 2.74 ) | **<0.001** | **<0.001** | 2.74 | ( 2.31 - 3.26 ) | **< 0.001** |
| **Do you think check-ups are recommended** | | | | | | | |
| Yes (reference) | 1 | | | | | | |
| No | 0.27 | ( 0.18 - 0.4 ) | **<0.001** | **<0.001** | 0.15 | ( 0.1 - 0.21 ) | **< 0.001** |
| I don't know | 0.46 | ( 0.25 - 0.86 ) | **0.015** | **<0.001** | 0.36 | ( 0.2 - 0.65 ) | **< 0.001** |

Multivariable model for the binary response "Yes, expects check-ups "vs "No, does not expect check-ups", ", adjusted for having an opinion about check-ups, importance or regularly checking one's health status and thinking check-ups are recommended.
McFadden Pseudo R squared = 0.1792
OR: Odds Ration, CIs: Confidence Interval
Right column shows unadjusted univariable restults for the same variables.

**Table S1.D. Perceived health status association with check-up expectations**

| Perceived health status as predictor for check-up expectations | | | |
|---|---|---|---|
| | Expects to have check-up examinations (unadjusted) | | |
| **Variable** | **OR** | **95% CI** | ***P* value** |
| **Perceived health status** (per 10 points) | 0.93 | ( 086 - 0.1 ) | 0.044 |

**Table S1.E. Predictors correlations**

**Correlation matrix among all models pedictors**

| | | 1 | 2 | 3 | 4 | 5 | 6 | 7 | 8 | 9 | 10 | 11 | 12 | 13 | 14 | 15 |
|---|---|---|---|---|---|---|---|---|---|---|---|---|---|---|---|---|
| 1 | Check-up | 1.00 | | | | | | | | | | | | | | |
| 2 | Sex | 0.07 | 1.00 | | | | | | | | | | | | | |
| 3 | Age group | -0.13 | 0.01 | 1.00 | | | | | | | | | | | | |
| 4 | Language | 0.07 | -0.02 | -0.01 | 1.00 | | | | | | | | | | | |
| 5 | Area type | 0.01 | 0.03 | 0.03 | -0.20 | 1.00 | | | | | | | | | | |
| 6 | Civil status | -0.09 | 0.10 | 0.63 | 0.00 | 0.01 | 1.00 | | | | | | | | | |
| 7 | Employment | 0.03 | 0.43 | 0.44 | 0.01 | 0.04 | 0.22 | 1.00 | | | | | | | | |
| 8 | Education | 0.01 | -0.06 | -0.10 | 0.19 | -0.13 | -0.03 | -0.09 | 1.00 | | | | | | | |
| 9 | Last check-up | 0.30 | -0.11 | -0.27 | -0.17 | 0.01 | -0.18 | -0.19 | -0.05 | 1.00 | | | | | | |
| 10 | Last GP visit | 0.07 | -0.05 | -0.23 | 0.04 | -0.17 | -0.11 | -0.26 | 0.12 | 0.27 | 1.00 | | | | | |
| 11 | Opinion | 0.37 | -0.11 | -0.28 | 0.27 | -0.07 | -0.17 | -0.10 | -0.07 | 0.28 | 0.09 | 1.00 | | | | |
| 12 | Importance | -0.39 | 0.02 | 0.13 | 0.07 | -0.04 | 0.09 | 0.13 | -0.06 | -0.37 | -0.35 | -0.16 | 1.00 | | | |
| 13 | necessary | 0.38 | 0.02 | 0.06 | -0.16 | 0.02 | 0.07 | 0.06 | -0.05 | 0.28 | 0.10 | 0.14 | -0.40 | 1.00 | | |
| 14 | recommended | 0.42 | 0.07 | 0.05 | 0.08 | 0.00 | 0.04 | 0.11 | -0.01 | 0.17 | 0.04 | 0.26 | -0.38 | 0.63 | 1.00 | |
| 15 | health | 0.01 | -0.05 | -0.14 | 0.04 | 0.04 | -0.13 | -0.19 | 0.10 | 0.09 | 0.23 | -0.05 | -0.18 | 0.02 | 0.04 | 1.00 |

| | |
|---|---|
| check-up | check-up expectation |
| opinion | do you have a formed opinion about check-ups |
| importance | importane of regularlz cheching one's health |
| necessary | do you thing check-ups are necessary |
| recommeded | do you thing check-ups are recommended |
| heatlh | perceived health status |
