# Supplementary material for: Expectations about check-up examinations among Swiss residents: A nationwide population-based cross-sectional survey
Source: PLoS One. 2021 Jul 21;16(7):e0254700. doi: 10.1371/journal.pone.0254700 (PMC8294504; doi:10.1371/journal.pone.0254700)
Supplement: S2 Table — (PDF) [file pone.0254700.s003.pdf]

Table S2. Percentage of expected Interventions per age group and in total

Swiss guideline recommendations (EviPrev)	Survey sample expectations					
**Intervention**	**age group**	**n - expects the test**	**% within age group**	**lower 95% CI**	**upper 95% CI**	**Stats**
**Counselling on tobacco** (n=579) Recommended to all ages	18 to 29	44	36	27.15	44.15	Chi=10.77 *P*=0.013
	30 to 44	51	33	25.88	40.77	
	45 to 59	43	21	15.49	26.77	
	60 to 99	26	25	16.76	33.64	
	total	163	28	24.48	31.81	
**Counseling on alcohol** (n=574) Recommended to all ages	18 to 29	47	39	29.96	47.16	Chi=17.97 *P*<0.001
	30 to 44	58	38	30.20	45.51	
	45 to 59	42	21	15.46	26.88	
	60 to 99	24	24	15.47	32.20	
	total	171	30	26.11	33.60	
**Counselling on nutrition** (n=577) If BMC > 27 kg/m2 + CV RF Recommended with counselling on pysical activity.	18 to 29	69	55	46.37	63.79	Chi=26.47 *P*<0.001
	30 to 44	86	55	46.95	62.56	
	45 to 59	75	38	30.89	44.37	
	60 to 99	27	28	19.32	37.24	
	total	257	44	40.43	48.54	
**Counselling on physical activity** (n=575) If BMC > 27 kg/m2 + CV RF Recommended with counselling on nutrition.	18 to 29	73	59	50.48	67.84	Chi=29.21 *P*<0.001
	30 to 44	82	52	43.96	59.57	
	45 to 59	67	34	27.59	40.89	
	60 to 99	31	31	22.32	40.63	
	total	252	44	39.84	47.96	
**Counselling on sexual health** (n=573) Recommended to the population at risk	18 to 29	77	62	53.18	70.28	Chi=61.79 *P*<0.001
	30 to 44	71	46	38.55	54.34	
	45 to 59	43	21	15.74	27.16	
	60 to 99	27	28	18.79	36.63	
	total	217	38	33.95	41.90	

Swiss guideline recommendations (EviPrev)	Survey sample expectations					
**Intervention**	**age group**	**n - expects the test**	**% within age group**	**lower 95% CI**	**upper 95% CI**	**Stats**
**low dose CT scan for lung cancer** (n=563) Between 55 and 80 y. (> 30 UPA, smoker or stopped smoking < 15 years ago)	18 to 29	74	62	53.66	71.08	Chi=2.68
	30 to 44	90	60	51.86	67.56	*P*=0.444
	45 to 59	106	54	46.76	60.70	
	60 to 99	55	56	46.22	65.95	
	total	324	58	53.47	61.64	
**Skin cancer prevention** (n=582) Not recommended	18 to 29	105	84	78.07	90.82	Chi=11.11
	30 to 44	128	80	74.27	86.60	*P*=0.011
	45 to 59	141	72	65.96	78.51	
	60 to 99	71	69	59.82	77.74	
	total	445	76	73.03	79.93	
**Colonoscopy** (n=573) From 50 y. > every 10 years	18 to 29	64	54	44.84	62.69	Chi=24.90
	30 to 44	110	72	64.80	79.03	*P*<0.001
	45 to 59	150	77	71.26	83.06	
	60 to 99	84	80	71.95	87.33	
	total	409	71	67.61	75.02	
**Faecal occult blood testing** (n=576) From 50 y. > every 2 years.	18 to 29	66	54	45.26	62.95	Chi=11.19
	30 to 44	103	65	57.42	72.26	*P*=0.011
	45 to 59	134	69	62.60	75.62	
	60 to 99	74	74	65.15	82.32	
	total	377	66	61.68	69.44	
**Blood test of PSA** (n=313) [M] Not recommended	18 to 29	41	67	55.40	78.88	Chi=10.61
	30 to 44	57	73	63.10	82.86	*P*=0.014
	45 to 59	96	85	77.99	91.24	
	60 to 99	52	86	77.13	94.72	
	total	246	79	74.01	83.10	
**Mammography** (n=276) [W] From 50 y. > every 2 years.	18 to 29	51	85	75.58	93.72	Chi=0.59
	30 to 44	68	86	78.85	94.02	*P*=0.898
	45 to 59	78	85	77.92	92.49	
	60 to 99	41	89	80.47	98.38	
	total	237	86	82.04	90.21	
**Papsmear test / cytology** (n=273) [W] Between 25 and 65 y. > every 3 years	18 to 29	52	85	76.59	94.33	Chi=4.07
	30 to 44	73	93	86.69	98.34	*P*=0.254
	45 to 59	75	83	75.20	90.71	
	60 to 99	36	82	70.63	93.45	
	total	235	86	82.01	90.22	

Swiss guideline recommendations (EviPrev)	Survey sample expectations					
**Intervention**	**age group**	**n - expects the test**	**% within age group**	**lower 95% CI**	**upper 95% CI**	**Stats**
**Vaccinations update** (n=578)<br>Recommended to all ages	18 to 29	92	74	66.07	81.48	Chi=10.28<br>*P*=0.016
	30 to 44	111	70	62.67	76.95	
	45 to 59	119	61	53.96	67.64	
	60 to 99	56	57	47.01	66.60	
	total	378	65	61.53	69.29	
**HIV screening** (n=562)<br>Recommended to the population at risk	18 to 29	89	71	63.20	79.07	Chi=79.91<br>*P*<0.001
	30 to 44	75	50	41.72	57.66	
	45 to 59	52	27	21.08	33.74	
	60 to 99	20	22	13.34	29.96	
	total	237	42	38.11	46.28	
**Depression** (n=560)<br>Recommended to all ages	18 to 29	69	57	47.94	65.53	Chi=46.29<br>*P*<0.001
	30 to 44	72	47	39.41	55.31	
	45 to 59	46	24	18.26	30.44	
	60 to 99	23	25	15.95	33.25	
	total	211	38	33.67	41.70	
**Blood test of lipid disorders** (n=589)<br>[M]> 35 y. and [W] > 45y. > every 5 years<br>With CV RF: individual	18 to 29	89	72	64.00	79.82	Chi=7.03<br>*P*=0.071
	30 to 44	119	76	69.03	82.44	
	45 to 59	150	74	67.84	79.92	
	60 to 99	90	86	78.97	92.40	
	total	448	76	72.61	79.50	
**Blood test of glucose** (n=583)<br>From 40 y.<br>With RF or BMI > 25 kg/m2: individual	18 to 29	89	73	64.70	80.45	Chi=6.58<br>*P*=0.087
	30 to 44	107	70	62.84	77.36	
	45 to 59	147	73	67.01	79.25	
	60 to 99	88	84	76.64	90.75	
	total	432	74	70.57	77.68	
**Blood pressure** (n=599)<br>Recommende to all ages	18 to 29	97	76	68.47	83.32	Chi=1.48<br>*P*=0.687
	30 to 44	130	82	75.50	87.53	
	45 to 59	159	78	72.05	83.45	
	60 to 99	85	79	71.64	86.97	
	total	471	79	75.36	81.92	
**Weight/obesity** (n=582)<br>Recommended to all ages	18 to 29	69	56	47.39	65.01	Chi=10.29<br>*P*=0.016
	30 to 44	81	51	43.38	58.96	
	45 to 59	80	40	33.60	47.25	
	60 to 99	42	41	31.31	50.24	
	total	272	47	42.66	50.76	

**Osteoporosis** (n=257) [W]<br>With RF: from 50 y.<br>From 65 y.	18 to 29	28	47	34.49	60.12	Chi=6.40<br>*P*=0.011
	30 to 44	42	56	45.06	67.59	
	45 to 59	48	59	48.27	69.62	
	60 to 99	31	73	59.81	86.44	
	total	149	58	51.86	63.93	
**Vision test** (n=590)<br>Not recommended	18 to 29	85	68	59.72	76.08	Chi=2.27<br>*P*=0.519
	30 to 44	95	61	53.18	68.45	
	45 to 59	120	60	53.44	67.02	
	60 to 99	69	64	54.78	72.89	
	total	370	63	58.77	66.58	
**Hearing test** (n=588)<br>Not recommended	18 to 29	72	57	48.47	65.80	Chi=1.41<br>*P*=0.703
	30 to 44	86	55	46.74	62.32	
	45 to 59	103	51	44.55	58.42	
	60 to 99	54	51	41.10	60.10	
	total	314	53	49.31	57.37	
**Electrocardiogram** (n=583)<br>Not recommended	18 to 29	89	70	61.92	77.85	Chi=1.58<br>*P*=0.665
	30 to 44	109	69	61.62	76.06	
	45 to 59	134	68	61.25	74.28	
	60 to 99	75	75	66.14	83.19	
	total	406	70	65.97	73.43	

Tables show the expectations for each medical intervention from all survey participants who either expected to have check-ups in addition to regular care and/or opportunistic prevention.
Number in parenthesis next to the intervention title is the number of people who responded yes/no to the expectation of the intervention.
Otherwise specified the intervention is meant for both men and women
If not otherwise specified, recommendations stop at 75 years old.
Legend:
eviprev = https://eviprev.ch/wp-content/uploads/2019/11/tableau_oct2016_f.pdf
CT = computed tomography, CV = cardiovascular, RF = risk factors, UPA = Unit package year
y. = years old, [M] = only men, [W] = only women
Chi = Chi-square test, *P* = *P* value
