# Supplementary material for: Expectations about check-up examinations among Swiss residents: A nationwide population-based cross-sectional survey
Source: PLoS One. 2021 Jul 21;16(7):e0254700. doi: 10.1371/journal.pone.0254700 (PMC8294504; doi:10.1371/journal.pone.0254700)
Supplement: S3 Table — (PDF) [file pone.0254700.s004.pdf]

Table S3. Swiss Population 2019

Swiss population 2019		
Total popualtion	8 606 033	
		Percentge
**Sex**	Male	49.6
	Female	50.4
**Age**	0-14	15.0
	15-19	4.9
	20-39	26.4
	40-64	35.0
	65-79	13.4
	80 or more	5.3
**Civil status** *(Calculated for adults aged 20 and over)*	Single	30.9
	Married	52.2
	Concubinage	0.3
	Divorced or dissolved partnership	10.7
	Widow	5.9
**Area type**	Urban	84.8
	Rural	15.2
**Languages** *(it is possible to indicate several main languages*)	German	62.7
	French	22.8
	Italian	8.3
	Romanche	0.5
	Others	22.4
**Employment** *(Population aged 15 and over)*	Self-employed	8.2
	Family members working on family business	1.2
	Employees	53.1
	Apprentices	2.7
	Unemployed	3.0
	In education or training	4.2
	Housewifes/househusbands	2.8
	Pensioners	22.4
	Other non-active people	2.4
**Education** *(People from 25 to 64 years old, 2020)*	Primary School	10.7
	Secondary School	35.5
	Professional School	8.5
	Middle School	15.1
	Technical School or University	30.1
Reference	Federal Statistical Office | Federal Statistical Office https://www.bfs.admin.ch/bfs/en/home.html *accessed:* 2021-06-01	

Otherwise stated the population distribution refers to year end 2019.
